# A biological circuit to anticipate trend

**DOI:** 10.1093/evlett/qrae027

**Published:** 2024-06-21

**Authors:** Steven A Frank

**Affiliations:** Department of Ecology & Evolutionary Biology, University of California, Irvine, CA, United States

**Keywords:** Evolutionary theory, plasticity, systems biology, financial models, stochastic time series

## Abstract

Organisms gain by anticipating future changes in the environment. Those environmental changes often follow stochastic trends. The steeper the slope of the trend, the more likely the trend’s momentum carries the future trend in the same direction. This article presents a simple biological circuit that measures the momentum, providing a prediction about future trend. The circuit calculates the momentum by the difference between a short-term and a long-term exponential moving average. The time lengths of the two moving averages can be adjusted by changing the decay rates of state variables. Different time lengths for those averages trade off between errors caused by noise and errors caused by lags in predicting a change in the direction of the trend. Prior studies have emphasized circuits that make similar calculations about trends. However, those prior studies embedded their analyses in the details of particular applications, obscuring the simple generality and wide applicability of the approach. The model here contributes to the topic by clarifying the great simplicity and generality of anticipation for stochastic trends. This article also notes that, in financial analysis, the difference between moving averages is widely used to predict future trends in asset prices. The financial measure is called the moving average convergence–divergence indicator. Connecting the biological problem to financial analysis opens the way for future studies in biology to exploit the variety of highly developed trend models in finance.

## Introduction

Predicting future environmental change provides many benefits. A microbe gains by anticipating the availability of sugars. A plant gains by forecasting the flow of nutrients. Plasticity benefits from a head start on altering physiology or form. Competitive players profit by preparing for the next step in a contest (DeWitt & Scheiner, 2004; Goo et al., 2012; Kussell & Leibler, 2005; Mitchell et al., 2009; Pfennig, 2021; Pigliucci, 2001; Reed et al., 2010; Shemesh et al., 2010; Siegal, 2015; Venturelli et al., 2015).

Organisms often anticipate regular patterns of change, such as circadian rhythms or seasonal cycles. However, many regularities arise as stochastic trends. To take advantage of such trends, a biological circuit must measure past directionality and use that measurement to predict the direction of future change.

In the literature, studies of *Escherichia coli* chemotaxis develop the most compelling models for the anticipation of trend (Alon, 2019; Shimizu et al., 2010; Tu et al., 2008). Cells measure changes in chemical concentrations to predict whether future changes will be increasing or decreasing. Tjalma et al.’s (2023) excellent recent article synthesizes past literature and develops new models.

This article introduces a simple model for anticipating trend. Roughly speaking, the model estimates the momentum of the current trend by the difference between a shorter-term moving average and a longer-term moving average. That estimate of momentum predicts the future direction of change because the future trend often continues in the direction of the current momentum.

Most existing models, such as those for *E. coli* chemotaxis, also base predictions on estimates of current or past trend. However, those previous models typically add specific aspects of a particular application or additional complexities, such as noise filtering. Those additions are interesting but also obscure the simplicity and generality of the underlying way in which estimates of momentum anticipate future trend.

The model here strips nonessential features to emphasize the fundamental structure of anticipation for simple stochastic trends. That abstraction provides the basis for future applications across a broader range of biological problems.

Empirically, the model predicts the primary mechanism that organisms use to anticipate environmental trends and the pattern of anticipatory response to environmental changes. Theoretically, the model provides the foundation for developing further predictions about tradeoffs between rapid adjustment of anticipated environmental changes and the susceptibility to perturbation of the circuit that predicts trends (Tjalma et al., 2023).

The model’s simplicity also reveals a close connection to the moving average convergence–divergence (MACD) indicator, the most widely used measure of momentum and trend to analyze asset prices in financial time series (Murphy, 1999; Pring, 2014; Schwager, 1999). That connection between biological models and financial analysis encourages application of the highly developed models of information and anticipation in finance to biological problems.

## The challenge

Let $u_{t}$ be a randomly varying input signal. We wish to predict the direction of change at a future time, t+1, relative to the current time, t, which means predicting the sign of $\Delta \,u_{t}=u_{t+1}-u_{t}$. If u changes in a purely random way, as a random walk with no directionality, then expected prediction success above 1/2 is not possible. For prediction to be possible, we must assume some pattern to the fluctuations in $u_{t}$.

**Figure 1:**
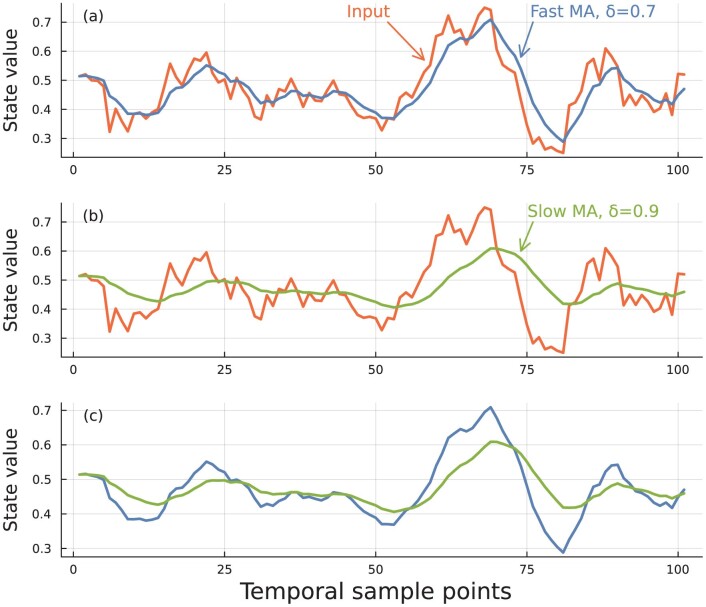
Relation between random input and exponential moving averages. (A) The input is generated by a continuous random walk, $\text{d}\,\hat{u}=0.2\,\text{d}\,W$, in which W is a Wiener process. The continuous process is sampled at time steps of 0.1 over 10 time units to yield 100 sample points. The Fast MA curve is an exponential moving average of the input. (B) The Slow MA curve shows a slower exponential moving average of the input. (C) Comparison of faster (blue) and slower (green) moving averages of the input.

An exponential moving average of purely random inputs is perhaps the most generic type of trend. Start with a random walk, ${\hat{u}}_{t}$, sampled at discrete times, t=0,1,…. Then replace each value at time t by its exponential moving average, $u_{t}=\left(1-\delta \right)\,{\hat{u}}_{t}+\delta \,u_{t-1}$. The value of 0≤δ≤1 sets the memory scale, with larger values averaging over longer time periods. Here, we linearly interpolate between the discretely sampled points to obtain values of $u_{t}$ continuously with time. Figure 1 illustrates random input and exponential moving averages with different memory scales.

The future change in $u_{t}$ is positive when


$$\Delta \,u_{t}=\left(1-\delta \right)\,\Delta \,{\hat{u}}_{t}+\delta \,\Delta \,u_{t-1}>0.$$ (1)

Because ${\hat{u}}_{t}$ is a random walk, its change is equally likely to be positive or negative. Thus, the best prediction for the sign of $\Delta \,u_{t}$ is the sign of $\Delta \,u_{t-1}$. In other words, the most recently observed direction of change provides the best prediction for the next direction of change.

Roughly speaking, we may think of the currently observed change, $\Delta \,u_{t-1}$, as the trend momentum. Positive momentum means that the trend is likely to continue up. Negative momentum means that the trend is likely to continue down.

The greater the momentum’s magnitude, the more likely the trend will continue in the same direction. For example, the larger $\Delta \,u_{t-1}$, the more negative the underlying random walk change, $\Delta \,{\hat{u}}_{t}$, must be to reverse the trend. Increasingly extreme moves in the underlying random walk are increasingly uncommon.

Thus, an ideal internal model uses the currently observed trend direction to predict the next direction of change. And it uses the trend momentum to estimate the confidence in the directional prediction.

## Exponential moving average

I claimed that an exponential moving average provides a common type of trend. I made that claim because any process that balances a fluctuating input against a steady decay describes an exponential moving average. For example, the differential equation


$$\dot{z}=u-\lambda \,z$$ (2)

determines a level of z that balances production or input, u, against steady decay at rate λ. With $u_{0}=0$, the value of z at time t is


$$z\,\left(t\right)=\int_{0}^{t}e^{-\lambda \,\left(t-\tau \right)}\,u\,\left(\tau \right)\,\text{d}\,\tau ,$$

which is proportional to the continuous time exponential moving average of z at that time. The process in Equation 1 is a discrete time version of the balance between input and decay.

The form of Equation 2 describes what is perhaps the most common expression of cellular biochemistry. The concentration of a molecule z balances the production, u, against the intrinsic decay rate, λ. Often one uses an additional parameter, α, to allow tuning of the production rate, as in the following section. In general, we may consider Equation 2 as an expression of any signal that balances external stimulation, u, and intrinsic decay, λ, including neural or physiological signals, or more abstract signals tracked by players in an evolutionary game.

## The circuit

To predict the trend direction, the following circuit calculates the difference between two moving averages, x and y, each average taken over a different time scale


$$\begin{array}{cc} \dot{x} & =\alpha \,u-\beta \,x\\ \dot{y} & =\gamma \,\left(\alpha \,u-\beta \,y\right). \end{array}$$ (3)

**Figure 2:**
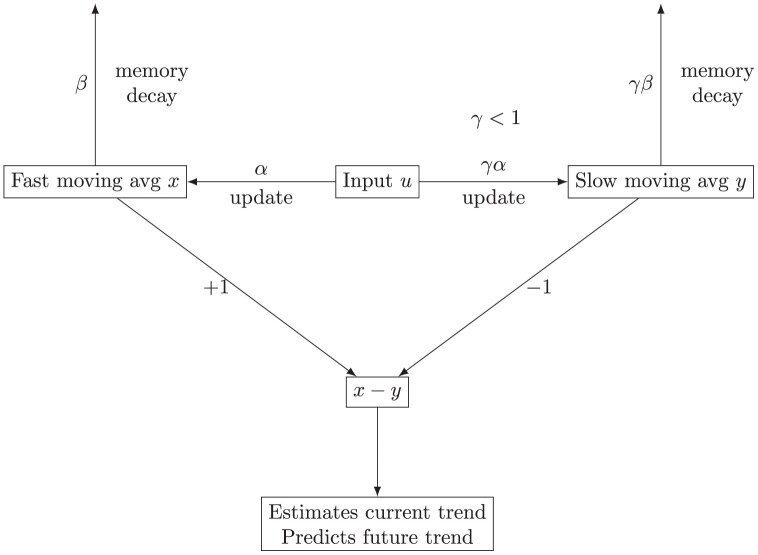
Illustration of the circuit in Equation 3. The input, u, updates the fast and slow moving averages, x and y. Those averages decay in relation to their memory scales, β and γ⁢β, respectively, with γ<1. The difference between the fast and slow moving averages estimates the recent trend and predicts the direction of change in the next time period.

Overdots denote derivatives with respect to time, t, and x,y,u are functions of time. For constant input, u, the molecular abundances of x and y have the same equilibrium values, α/β. As the input, u, changes, y changes more slowly than x when γ<1. Thus, x−y tends to be positive when u is increasing and negative when u is decreasing. The values of x and y are approximately proportional to shorter and longer exponential moving averages of the input, u. Figure 2 illustrates the circuit.

I have presented this model abstractly to highlight generic aspects of process. With regard to application, the magnitude of a biological signal often arises from the opposition of production and decay. In such cases, the parameters of production, α, and decay, β, may be estimated empirically from data or evaluated theoretically for their phenotypic consequences. The parameter γ scales the relative production and decay rates between two related signals.

## An example

We can optimize the parameters relative to a particular goal. In an evolutionary context, natural selection often tends to change systems in the direction indicated by an optimality analysis (Maynard Smith, 1978). Thus, we can gain clues about how natural selection may tune mechanistic aspects of systems in order to predict future trends.

**Figure 3:**
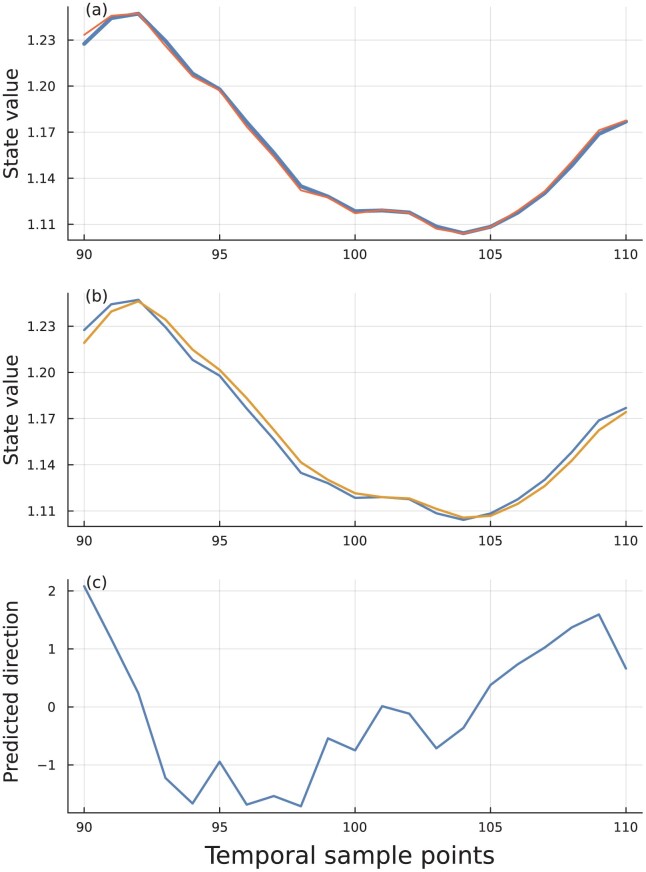
Prediction for the direction of change in a sequence of observations. Dynamics given by Equation 3. In this example, the parameters α,β,γ were optimized for accuracy of prediction by the Nelder–Mead method, yielding 0.1663,0.08314,0.2782, respectively. The plots show a subset of time points to magnify patterns and make them easier to see. (A) The faster of the two moving averages, x, in the thicker blue curve tracks the input sequence, $u_{t}$, in thinner red curve. (B) The fast and slow moving averages, x and y, in with the fast blue curve above the slow gold curve at time points 90 and 110. The fast blue curves in this panel and the previous one are the same. (C) The difference between the moving averages, plotted as 1000⁢[σ⁢(x−y)−0.5], in which $\sigma \,\left(z\right)=e^{z}/\left(1+e^{z}\right)$ is the sigmoid function. The sign predicts the direction of change in the next time step. The magnitude reflects the momentum, a measure of the relative confidence in the prediction for the future direction of change. To calculate the input sequence in Equation 1, the random walk follows a Wiener process with mean 0 and standard deviation 0.2, and the exponential moving average memory parameter is δ=0.2. For each realized sequence of the random walk, the values are normalized to [0.25,0.75] by affine transformation, yielding $\hat{u}$. The timescale and abundances in the plots have arbitrary units. The freely available computer code describes the scaling of time, the parameters, the optimization, and the production of graphics (Frank, 2024).

Figure 3 shows an example of an optimized circuit that predicts the future direction of change in $u_{t}$. Define maximum potential accuracy as the frequency at which the prior sign of change in $u_{t}$ predicts the next sign of change, a continuation of trend.

In Figure 3, the maximum potential accuracy is 0.8, measured by the match between the signs of sequential differences in long input sequences. Stochasticity in the input sequence means that sometimes the sign of the prior difference does not match the direction of the next difference. For this example, the optimized circuit accuracy is close the maximum potential accuracy, with a median deviation from the maximum of 0.001, calculated as the difference of the actual match between sequential differences in the input and the circuit’s success is predicting the sign of each future difference along the inputsequence.

Figure 3B shows the classic MACD pattern for trend prediction from financial analysis. The slower signal in gold, which is the variable y, lags the faster signal in blue, which is the variable, x. A predicted trend reversal arises when the fast blue signal crosses the slow gold signal, that is, when the sign of x−y changes. Figure 3C traces a function of the x−y difference.

A convergence between the trends in Panels B and C predicts a continuation of the current trend. A divergence between the trends in the two panels foreshadows a potential upcoming change in direction for the trend.

For example, starting at time point 98, the x−y difference begins to shrink, causing an upturn in the trend in Panel C. That uptrend signals a slowing of the downward momentum. At first the actual trend in Panel B continues downward. Thus, the trends in the two panels are diverging. Then, at time point 104, the actual trend in Panel B turns up, a convergence with the trend in Panel C, signaling the potential start of an upswing, which in fact does occur.

Typically, a change in momentum (Panel C) precedes a change in trend (Panel B), as happens around time point 104. The reason a slowing of momentum typically precedes a change in trend can be seen in Equation 1, which shows how changes in the direction of an exponentially smoothed input are often dominated by the momentum term. However, when the trend changes abruptly, momentum does not precede trend, as happens around time point 92.

## Accuracy vs. robustness

High accuracy requires that the x−y predictor in Figure 3C switches sign immediately when the input turns down. The input appears as the red line in Figure 3A. To achieve high accuracy in forecasting a change in trend direction, in Figure 3B, the slower moving average, y, in gold, must remain close to the faster moving average, x, in blue.

The small difference between the moving averages, x and y, means that false signals can arise from small perturbations. For example, at time point 102 in Figure 3B, the moving averages touch, predicting an imminent reversal that does not subsequently occur. In addition, noise in the state variables x and y can trigger false signals.

**Figure 4:**
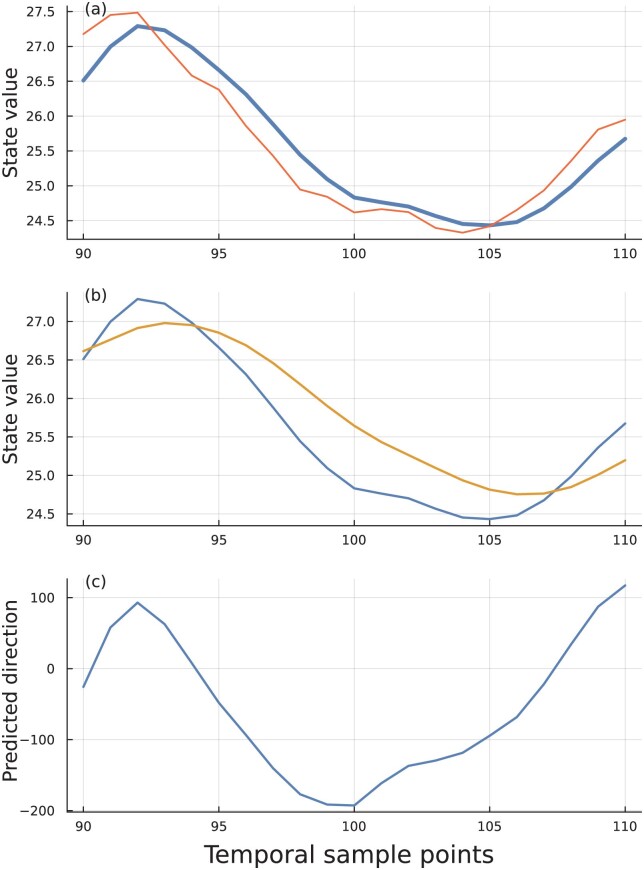
Tradeoff between accuracy and robustness. Same plots as Figure 1 but with slower moving averages. Optimized parameters α,β,γ are 0.4179,0.009483,0.2587, respectively.

Figure 4 shows the circuit optimized for wider differences between the fast and slow moving averages, x and y. In Panel B, the wider differences provide a stronger signal during a trend but lag in giving a signal when the trend does change direction. This system gives fewer false signals and is robust to small perturbations. However, because it lags when the trend does change, it has a lower accuracy when using the sign of the current difference, x−y, to predict the direction of change in the input in the next time period. The median deviation from the maximum accuracy is 0.12, compared with a deviation of 0.001 in the circuit illustrated in Figure 3.

In general, systems can be tuned to balance various tradeoffs, such as accuracy vs. robustness (Murphy, 1999; Pring, 2014; Schwager, 1999; Tjalma et al., 2023). In evolution, problems of perception often must distinguish between changes in trend vs. fluctuations caused by noise. If a system alters its internal signal estimate too rapidly in response to noise, then many predictions about trend will be false. If it changes too slowly, then it will lag real changes in trend. Examples include cellular chemotaxis, signals of attack, and physiological tracking of environmental state. The analysis here concerns how natural selection will tune mechanisms of perception and signal estimation in relation to those widespread challenges of life.

## Conclusion

Prior models analyzed how biochemical circuits predict future environmental changes (Alon, 2019; Becker et al., 2015; Mitchell et al., 2009; Shimizu et al., 2010; Tjalma et al., 2023; Tu et al., 2008). Those models use recent differences in input to predict future changes because that is the essential nature of the problem.

Although the prior models described biological circuits that predict environmental changes, none of those prior analyses presented a circuit and an explanation as simple as those given here. In essence, the difference between a shorter, more immediate moving average, x, and a longer, slower moving average, y, provides the basis to forecast trends.

In the technical analysis of financial prices, the MACD indicator for moving average convergence-divergence is calculated as the difference between longer and shorter moving averages. Organisms may use a similar calculation to anticipate environmental trends.

## Data Availability

Software code and output for all analyses and figures available at GitHub (Frank, 2024).
